# Evaluation of nocturnal vs. morning measures of heart rate indices in young athletes

**DOI:** 10.1371/journal.pone.0262333

**Published:** 2022-01-05

**Authors:** Christina Mishica, Heikki Kyröläinen, Esa Hynynen, Ari Nummela, Hans-Christer Holmberg, Vesa Linnamo

**Affiliations:** 1 Faculty of Sport and Health Sciences, University of Jyväskylä, Jyväskylä, Finland; 2 KIHU–Research Institute for Olympic Sports, Jyväskylä, Finland; 3 Department of Health Sciences, Luleå University of Technology, Luleå, Sweden; University of Bourgogne France Comté, FRANCE

## Abstract

**Purpose:**

The purpose of this study was to compare heart rate (HR) and heart rate variability in young endurance athletes during nocturnal sleep and in the morning; and to assess whether changes in these values are associated with changes in submaximal running (SRT) and counter-movement jump (CMJ) performance.

**Methods:**

During a three-week period of similar training, eleven athletes (16 ± 1 years) determined daily HR and heart rate variability (RMSSD) during sleep utilizing a ballistocardiographic device (Emfit QS), as well as in the morning with a HR monitor (Polar V800). Aerobic fitness and power production were assessed employing SRT and CMJ test.

**Results:**

Comparison of the average values for week 1 and week 3 revealed no significant differences with respect to nocturnal RMSSD (6.8%, P = 0.344), morning RMSSD (13.4%, P = 0.151), morning HR (-3.9 bpm, P = 0.063), SRT HR (-0.7 bpm, P = 0.447), SRT blood lactate (4.9%, P = 0.781), CMJ (-4.2%, P = 0.122) or training volume (16%, P = 0.499). There was a strong correlation between morning and nocturnal HRs during week 1 (r = 0.800, P = 0.003) and week 3 (r = 0.815, P = 0.002), as well as between morning and nocturnal RMSSD values (for week 1, r = 0.895, P<0.001 and week 3, r = 0.878, P = 0.001).

**Conclusion:**

This study concluded that HR and RMSSD obtained during nocturnal sleep and in the morning did not differ significantly. In addition, weekly changes in training and performance were small indicating that fitness was similar throughout the 3-week period of observation. Consequently, daily measurement of HR indices during nocturnal sleep provide a potential tool for long-term monitoring of young endurance athletes.

## Introduction

Over the past several decades, the scientific approach to finding a balance between endurance training and recovery has continued to grow [1], resulting in the application of various tools and methods to monitor athlete recovery [2]. Since the outcome of an endurance competition is often influenced by relatively small differences in performance, tests of performance, such as submaximal running (SRT) and counter-movement jump (CMJ) tests, are commonly utilized for monitoring athletes’ current physical condition [3]. For instance, during a two-week period with an increasing training load, the submaximal lactate levels observed during a SRT declined, with a subsequent return to normal following two weeks of recovery [4], demonstrating that physiological changes are reflected rapidly in the results of a SRT. In addition, decreases in the submaximal heart rate (HR) and/or increases in heart rate variability (HRV), measured using the root-mean-squared difference between successive RR intervals (RMSSD), for young soccer players as their training season progressed most likely reflect improvements in performance [5], indicating that these values might be of use for monitoring young athletes.

Recovery from endurance training involves a multitude of physiological responses, with the cardiovascular system playing a key role in this context [6, 7]. This process is regulated by the autonomic nervous system (ANS) and the time required for autonomic recovery is an indicator of cardiovascular homeostasis [7, 8]. Autonomic cardiovascular function can be assessed non-invasively by measuring HRV, i.e., the beat-to-beat variation in heart rate [9, 10] and advances in technology have provided affordable and reliable means for monitoring HRV and its response to daily stress [10]. Consequently, use of this approach, both for practical and research purposes, has become increasingly common [11].

HRV is regulated by the sympathetic and parasympathetic activity of ANS, and two common methods for measuring HRV are time and frequency domain [12]. RMSSD measures are a time domain method where sympathetic activity decreases the time between heartbeats while increases are due to parasympathetic activity [10]. During rest, the ANS favors parasympathetic activity [7], therefore an easy and practical way to monitor HRV is to follow resting RMSSD values [13]. There is considerable variation in procedures concerning what HRV methods are the best compromise between quality and accuracy of recordings as well as ease of use for athletes [13]. Sport technology devices are not specifically designed for research application, but validation of the recorded data is critical. Developments in technology with heart rate monitor devices have shown to produce recordings of RR intervals consistent to electrocardiograph (ECG) recordings and HRV parameters resulting from these recordings are comparable for performing orthostatic tests in healthy subjects [10]. Furthermore, mixed results in previous research indicate that HRV alone may not provide a comprehensive view of an athletes’ overall wellbeing [2]. However, when used in combination with performance tests, and/or training data and questionnaires these values appear to be some of the most valuable variables to monitor [2].

Therefore, the purpose of this study was to access the ability of nocturnal HR and RMSSD values determined by ballistocardiography (BCG) to predict the respective validated morning values recorded with a heart rate monitor during orthostatic tests [10]. The aim was to compare HR and RMSSD values to provide results that can reflect the agreement between the two methods with additional SRT and CMJ tests to follow changes in physical performance during the 3-week training period. It was hypothesized that nocturnal and morning weekly HR and RMSSD values would show agreement, but morning orthostatic values would have a slightly lower RMSSD and slightly higher HR than nocturnal values due to additional thoughts and disturbances that may affect autonomic regulation when awake [14]. Secondly, it was hypothesized that changes in RMSSD and HR would be related to fatigue and therefore, associated to performance changes in SRT and CMJ tests. Finally, if weekly agreement between nocturnal and morning HRV tests was present, the goal was to determine if nocturnal measures are a valid method for monitoring young endurance athletes.

## Methods

### Participants and design

Eleven well-trained young endurance athletes participated in this study. All participants were students at a sports academy high school competing and training for cross-country skiing (10 subjects) and biathlon (1 subject) year-round. Characteristics of the athletes are presented in Table 1. All were fully informed of the experimental procedures and provided written consent from their legal guardians before taking part. The ethics committee of the University of Jyväskylä, Finland, approved the study and measurements were performed in accordance with the declaration of Helsinki.

**Table 1:** Characteristics of the subjects (means ± SD).

	Women (n = 4)	Men (n = 7)
**Age (yrs)**	16 ± 1	16 ± 1
**Height (m)**	1.67 ± 0.09	1.79 ± 0.02
**Body mass (kg)**	61 ± 10	68 ± 5
**Body fat (%)** ^**a**^	17.6 ± 1.5	7.9 ± 2.3
**BMI (kg.m** ^**-2**^ **)**	21.6 ± 1.3	21.1 ± 1.3

^a^ Assessed on the basis of bioimpedance measurements.

**Fig 1:**
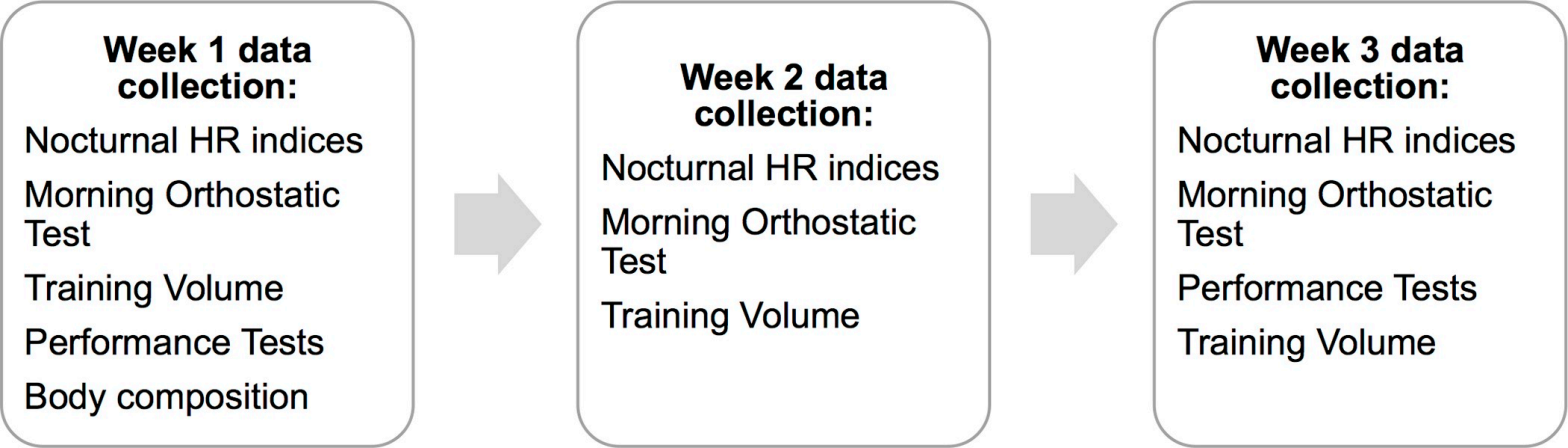
Flow chart of study design.

This study occurred during a 3-week period of normal endurance training in preparation for the upcoming racing season (early November). Preceding participation, practice tests were provided so subjects were familiarized with the CMJ and SRT protocols. Running and jump exercises were already incorporated into the tested individuals’ normal training so a preparatory training period was unnecessary. The SRT and CMJ tests were performed during week 1 and week 3 of the study. During night sleep, ANS state was assessed with nocturnal HRV and HR analysis, collected using a ballistiocardiographic (BCG) sleep-tracking device (Emfit QS, Emfit Oy, Jyväskylä, Finland). In addition, morning values for HRV and HR were also evaluated with orthostatic tests performed using a Polar V800 heart rate monitor (Polar Electro Oy, Kempele, Finland) and H10 heart sensor (Polar Electro Oy, Kempele, Finland). Morning and nocturnal values for HR and HRV were measured daily throughout the study. At the beginning of the study, body fat percentage and weight were measured using the bioimpedance method (InBody 720, Inbody CO., Cerritos, California, USA). Fig 1 illustrates the study design and when each measurement occurred.

### HRV and HR analysis

Nocturnal HR and HRV were monitored using a contactless sleep-tracking device. This device employs BCG to numerically and graphically depict repeated movements [15], such as heartbeat, with a contactless pressure (542 mm x 70 mm x 1.4 mm) and then presents the values numerically. Under free-living conditions, evaluation of this device revealed good agreement to a laboratory validated reference device utilizing electrocardiography, with only minor differences in mean HR and HRV values [16]. Therefore, although the accuracy of this device for continuous monitoring of nocturnal HRV and HR is yet to be determined, it seems to be an effective tool and convenient method for automatic analysis of HR and HRV.

Subjects were instructed to place the device under the mattress near the chest area to minimize the distance to the heart and thus, maximize the signal quality. The subjects were unable to detect the device’s presence, but the device sensed when body weight was present and automatically began the recording process. The recording continued at a sampling rate of 100 Hz, stopping when the subject left the bed in the morning. Nocturnal HR and HRV data were collected using continuous 3-minute periods, and disrupted and/or poor signals were excluded from the data. The average HR values for each 3-minute period were calculated and these averages were used to determine whole night daily averages. Nocturnal HRV was interpreted relative to time, by utilizing RMSSD. To determine several different nocturnal RMSSD values during each night, average RMSSD for each 3-minute period was calculated and used to produce a graphical representation of nocturnal RMSSD values. The endpoints of the best linear fit for each night were chosen to represent the average RMSSD values for evening (RMSSD_pm_) and morning (RMSSD_am_) sleep and the whole night average (RMSSD_NOC_) was considered to be the mean of both values. Although previously the average 3-minute values for the entire night have been used for analysis [16], the current investigation focused on using values that are automatically presented on the user interface of the sleep device. Values presented there were obtained from approximately 8–10 hours of sleep each night.

Morning orthostatic tests were performed using a Polar V800 heart rate monitor and H10 heart sensor. This sensor has previously shown acceptable levels of agreement with a 12-lead electrocardiogram system for recording RMSSD [17] and the Polar V800 monitor is a previous validated method for detection of RR intervals during an orthostatic test [10]. All subjects were instructed to keep the heart rate monitor and electrode strap next to their bedside and to conduct the orthostatic test upon waking up each morning. This assured minimal physical activity occurred. No attempts were made to control the breathing frequency, and subjects were asked to remain relaxed and repeat the test using the same routine each morning. Instructions were provided in the orthostatic test feature of the Polar V800 watch with the test beginning at the click of a button. Subjects were directed to remain in a supine position for 3 minutes before a beeping occurred from their watch signifying that the subject needed to stand up. The standing portion of the test was also 3 minutes in length with a beep indicating the test was complete. Orthostatic tests measures HR and HRV in R-R intervals using a sampling frequency of 1000 Hz with a reference window of 60–120 seconds in each position. Collected data was analyzed with polar flow (www.flow.polar.com). Test results provide average values for HR and HRV in both the supine and standing position as well as peak HR when standing. Minimal compliance for HRV and HR analysis was set at 3 days each week, therefore, if a subject did not have 3 days in one of the test weeks they were omitted from the study.

### Submaximal running test and counter movement jumps

Counter movement jump (CMJ) performance tests [18] were evaluated using a force plate (HUR FP8, HUR Oy, Kokkola, Finland). Subjects were instructed to keep their hands fixed to their hips, feet shoulder-width apart and to bend their knees to a 90-degree angle when jumping as high as possible. A total of three jumps were performed with about 1 minute of recovery between jumps. Jumping height was analyzed from the force impulse. The analysis was completed using coach tech system (Vuokatti Sports Technology Unit, University of Jyväskylä, Finland) [19]. The highest jump was used for the current measure of performance.

SRT tests were conducted to evaluate aerobic fitness. The SRT performed during this study was designed to elicit heart rates (HR) at approximately 90% of maximal HR so significant changes in HR variation were measurable [20]. The SRT included 4 stages and was 16-minutes in length. The test was performed on a Tunturi GO Run 50 Treadmill (Tunturi Fitness, Flevoland, Netherlands) and speed was standardized (women: 10.0 km/h, men: 11.7 km/h) with inclination increasing every 4 minutes, starting at 2%, then 4%, 7%, and 9%. The subjects’ HR was continuously monitored with a Polar HR-monitor and when 15 seconds of each load remained HR values were recorded. Every 4 minutes the subjects briefly stop running and blood samples (20 μL) were taken from the fingertip to determine blood lactate concentrations (Biosen C_line Lactate Analyzer, EKF Diagnostic, Magdeburg, Germany). Sample collection time (approx. 15 s) was included in the 4 minutes of the upcoming stage. Due to the highly homogenous nature of the group, the utilized protocol was appropriate and submaximal intensities were reached for all subjects. Although we do not have validation of this specific protocol, it is a familiar and standardized protocol that is commonly used as a control test by junior cross-country skiers and biathletes in Finland. Performance test measures occurred during week 1 and week 3 of the study period and measurements were required to be completed by all subjects each test week.

### Statistical analysis

Descriptive statistics were calculated for all variables and all values are reported as means ± SD with the 95% confidence interval (CI). Sample distribution was tested using the Shapiro-Wilk test for nocturnal and morning HR indices (i.e., HR and RMSSD) as well as performance test variables. Normal distribution was present for all weekly variables as well as the difference between weeks for nocturnal and morning HR indices. A within-subject approach was applied by utilizing paired sample t-tests to examine the differences between nocturnal versus morning measurements and the differences between the different test weeks. To evaluate the extent of agreement between morning and nocturnal values Pearson’s correlation coefficient (r) was calculated. To assess the difference between measurements made in the morning or during the night, the coefficient of variance and 95% CI for HR (HRCV) and RMSSD were determined each week for all HRV parameters (HRVCV).

The extent of correlation among the morning and nocturnal measures for HR and three different nocturnal HRV measures (AM, PM and Nocturnal) was tested using Intra-class correlation coefficient (ICC) or reliability coefficient, a measure of the reliability of measurements. ICC estimates and their 95% confidence intervals were calculated by a single-rating, consistency-agreement, 2-way mixed-effects model [21]. Values less than 0.5 suggest poor reliability with values between 0.5 and 0.75 suggesting moderate reliability and values between 0.75 and 0.9 indicative of good reliability [21].

## Results

**Fig 2:**
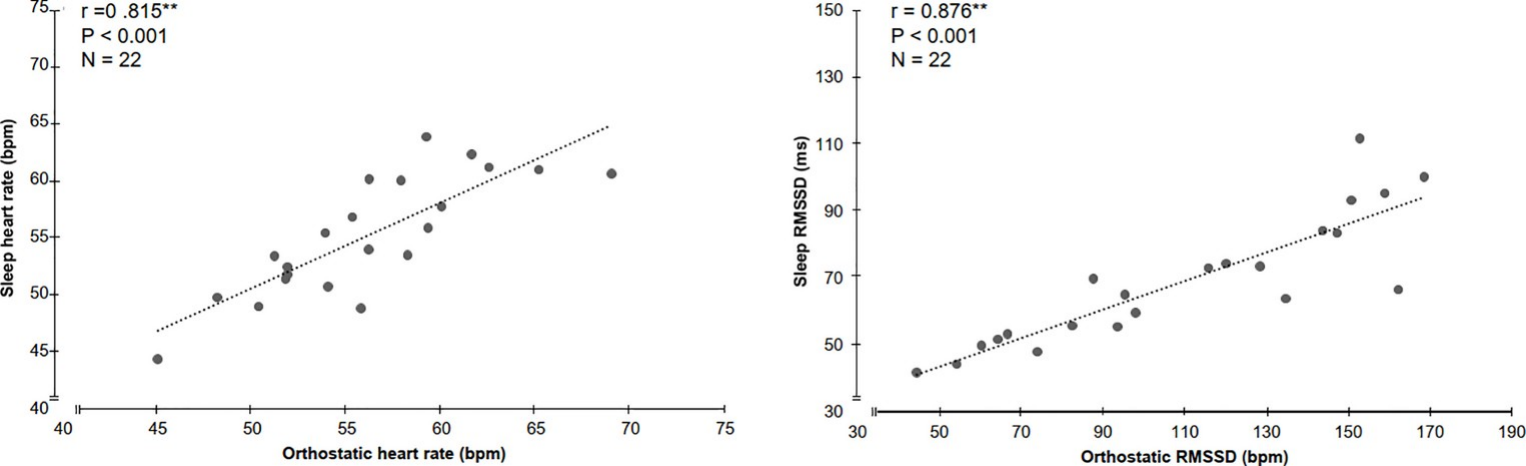
The relationship between nocturnal (sleep) and morning measurements for HR and RMSSD during the whole test period.

Table 2 shows weekly means and standard deviations of all measured variables. Paired samples t-tests revealed no significant differences between week 1 and week 3 for RMSSD_NOC_ (mean difference 6.8%, P = 0.344), morning RMSSD (mean difference 13.4%, P = 0.151), morning HR (mean difference -3.9%, P = 0.063), SRT HR (mean difference -0.7%, P = 0.447), SRT blood lactate (mean difference 4.9%, P = 0.781), CMJ (mean difference -4.2%, P = 0.122) or training volume (mean difference 16%, P = 0.499). Individual and group values for HR and RMSSD during morning and sleep are presented in Table 3. HR_CV_ and RMSSD_CV_ values are presented in Table 4 with HR presenting similar values in both morning and nocturnal measurements. Furthermore, no significant differences were found in HR between the measurements obtained during night sleep (Emfit QS) vs. morning orthostatic tests (Polar V800). During both test weeks, significant differences were found for RMSSD values between devices (P<0.008) and very high correlations were observed for RMSSD_NOC_ during week 1 (r = 0 .895, P<0.001) and week 3 (r = 0.878, P<0.001). Table 5 presents the IIC values of the measured HR indices. Moderate to good reliability was found for all HRV measures with nocturnal sleep and morning HR values showing the best agreement. Fig 2 displays the relationships for HR and RMSSD between nocturnal and morning measurements during the whole test period.

**Table 2:** Comparison of heart rate variability, heart rate, sleep, performance tests and training during the study period.

	Week 1		Week 3			
	mean ± SD	95% CI	mean ± SD	95% CI	% diff	P
**Nocturnal**						
RMSSD^AM^ (ms)	75 ± 26^a^	57.7, 92.7	77 ± 17^a^	65.6, 88.7	8.0	0.654
RMSSD^PM^ (ms)	60 ± 20^a^	46.7, 73.2	63 ± 21^a^	49.5, 77.2	6.6	0.306
RMSSD^NOC^ (ms)	68 ± 22^a^	52.6, 82.4	70 ± 17^a^	58.9, 81.5	6.8	0.344
Heart rate (bpm)	57 ± 5	53.0, 60.0	54 ± 5	50.2, 56.9	-5.2	0.002*
Sleep (hours)	8.2 ± 0.4	8.0, 8.5	8.6 ± 0.6	8.2, 9.0	3.8	0.095
**Morning Orthostatic**						
RMSSD^Rest^ (ms)	104 ± 39^a^	77.8, 130.2	115 ± 41^a^	87.1, 142.4	13.4	0.151
RMSSD^Stand^ (ms)	26 ± 8.6	18.3, 33.5	29 ± 9	19.9, 37.3	14.5	0.361
Heart rate (bpm)	57 ± 6	53.3, 61.3	55 ± 5	51.3, 58.5	-3.9	0.063
**Performance Test**						
Heart rate (bpm)	181 ± 6.9	176.3, 185.0	180 ± 8.2	174.0, 185.0	-0.2	0.447
Heart rate range (bpm)	28 ± 5.8	24.4, 32.2	30 ± 5.4	26.1, 33.3	1.3	0.181
Blood lactate (mmol/l)	3.6 ± 0.9	3.0, 4.1	3.6 ± 1.0	3.0, 4.3	0.4	0.781
Counter movement jump (cm)	34.1 ± 6.3	29.9, 38.4	32.5 ± 5.3	28.9, 36.0	-1.2	0.122
**Training**						
Volume (min/week)	514 ± 141	396.1, 632.4	563 ± 156	432.7, 693.0	16	0.499

* p < 0.01.

^a^ Significant difference between nocturnal and morning RMSSD_Rest_

**Table 3:** Descriptive statistics and coefficient of variance of individual and group weekly means ± SD for heart rate and RMSSD values during nocturnal sleep and morning tests.

	Average Heart Rate						Average RMSSD					
	Morning			Sleep			Morning			Sleep		
Subject	W1	W3	CV*	W1	W3	CV*	W1	W3	CV*	W1	W3	CV*
1	50	54	8.16	49	51	6.23	98	83	26.46	60	56	10.46
2	63	60	3.14	61	58	3.92	45	64	8.26	42	52	15.56
3	62	65	7.10	62	61	3.27	74	60	41.65	48	50	10.85
4	69	59	5.27	60	56	4.00	54	67	36.87	44	53	13.95
5	55	52	4.66	57	52	1.63	94	95	15.69	56	65	8.00
6	58	56	9.39	53	49	5.33	116	129	22.37	73	74	7.45
7	56	51	3.83	54	53	3.22	135	162	17.70	64	67	7.02
8	48	45	4.28	50	44	5.11	169	151	9.25	100	93	9.94
9	58	54	5.86	60	55	4.25	88	147	26.22	70	84	20.54
10	59	56	4.50	64	60	2.95	120	144	14.54	74	84	4.71
11	52	52	4.18	52	51	5.34	153	159	12.15	112	95	11.67
**Mean**	**57**	**55**	**5.49**	**57**	**54**	**4.11**	**104**	**115**	**20.01**	**68**	**70**	**11**
**SD**	**6**	**5**	**1.96**	**5**	**5**	**1.33**	**39**	**41**	**22.17**	**17**	**11**	**4.46**

**Table 4:** Coefficient of variance (CV) during nocturnal and morning heart rate measures.

	Week 1		Week 3	
	CV (%)	95% CI	CV (%)	95% CI
**Nocturnal**				
RMSSD^AM^ (ms)	16.20	10.99, 21.42	13.02	9.33, 16.71
RMSSD^PM^ (ms)	21.13	16.73, 25.46	21.37	15.43, 27.30
RMSSD^NOC^ (ms)	12.35	8.08, 16.63	9.49	7.33, 11.66
Heart rate (bpm)	4.17	2.88, 5.45	4.06	2.59, 5.53
**Morning**				
RMSSD^Rest^ (ms)	22.60	11.51, 33.68	19.43	9.43, 29.44
RMSSD^Stand^ (ms)	38.11	22.19, 54.02	39.96	22.00, 57.93
Heart rate (bpm)	5.78	3.57, 8.00	5.19	4.00, 6.39

**Table 5:** Intra-class correlation coefficient of morning versus nocturnal heart rate measures.

	Week 1			Week 3		
	ICC	95% CI	P	ICC	95% CI	P
**Nocturnal**						
RMSSD^AM^ (ms)	0.813	0.45, 0.95	0.001	0.534	0.06, 0.85	0.037
RMSSD^PM^ (ms)	0.691	0.20–0.91	0.006	0.650	0.12, 0.89	0.011
RMSSD^NOC^ (ms)	0.769	0.35, 0.93	0.002	0.617	0.06, 0.88	0.016
Heart rate (bpm)	0.793	0.40–0.94	0.016	0.810	0.44, 0.95	0.001

## Discussion

The main results from the current study were that HR and RMSSD values obtained with a BCG device under real-life conditions during sleep were in good agreement to morning values derived from orthostatic tests (Fig 2), providing support for our hypothesis that measurements in the morning with orthostatic tests as well as nocturnal measurements give reliable weekly values for the HR and RMSSD of young endurance athletes.

At the same time, our hypothesis that resting RMSSD and HR would be slightly lower and higher, respectively, than the corresponding nocturnal values turned out to be incorrect, since the former value obtained with orthostatic tests in the morning was higher and HR very similar at both time-points. Previous studies on the HRV of individuals with high levels of stress under real-life conditions revealed a lower variation in the values obtained with orthostatic tests after awakening, but not during nocturnal rest, suggesting that parasympathetic withdrawal occurs upon awakening [22]. In the case of our own investigation, in which none of the participants was in a stressed or over-trained state, the HR was similar at both time-points and the differences observed are likely to be due to the two different measurement procedures.

Previous research has challenged the evaluation of HR and RMSSD to monitor athletes, suggesting that the high day-to-day variation and variability of these values limits their usefulness because small and moderate changes often occur inside differences that are normally expected [23]. However, findings show that when comparing weekly vs. single day HRV values, the analysis of weekly HRV values provided a more meaningful assessment of ANS response in endurance athletes [24]. Additionally, longitudinal studies following RMSSD support this idea suggesting that weekly and rolling averages appear to have a more meaningful assessment of change in cardiac autonomic balance compared to isolated daily values [24]. As a result, using weekly mean values obtained from daily HRV recordings may improve the diagnostic utility of using HRV indices [24, 25]. Hence, the present study measured the daily HR and RMSSD values but utilized the weekly average values for further analysis.

Heart rate variability corresponds to countless stimulus and is influenced by physiological, psychological and environmental factors [26]. As a result, observable differences in nocturnal vs. morning RMSSD measures were present (Table 2). During sleep, individuals are no longer affected by external stimuli and therefore, enhanced reliability may exist for recordings that occur during sleep [14, 27, 28]. Contrary to the hypothesis, the current study showed significantly higher variation and higher RMSSD values during morning tests. In addition, slight differences in reliability between morning and nocturnal values was also present (Table 5). This may be the result of the difference in duration and/or time of day that were measured. Previous research has suggested that combining sleep periods into a single segment introduces a noise that reduces the detection of changes in HRV in over-trained athletes [29], which may also explain the differences in the present study. In addition, cardiac autonomic regulation was disrupted in HRV values, obtained after awakening, of over-trained athletes but not during night sleep [30]. However, this study did not investigate over-trained athletes and despite slight differences in weekly mean values, the nocturnal and morning RMSSD values showed strong relationships during both weeks. This finding suggests that when following weekly average values, measurements taken during nocturnal and morning rest are both acceptable. Although sleep values may be affected by movement and sleep stages, the use of weekly mean values has shown an increased ability to detect changes in performance [18], suggesting long-term weekly monitoring may be an effective tool for young athletes. Moreover, an additional advantage to the average values obtained during night sleep may be an increased ability to compare results. In previous research, the use of variation in body posture, time of day, sleep cycle and daily-vs.-weekly averages has presented disagreement in the direction of change in vagal-related HRV indices, which may be a result of methodological differences [2]. The use of whole night average values may help eliminate these differences and allow for comparison between studies.

Although coaches and athletes have followed HR for decades, current research has placed greater emphasis on HRV, considering it a more sensitive tool [2]. Research monitoring changes in HR during sleep found daily HR variation of about 8 bpm [31]. However, when observing the fatigue status of endurance athletes over a 4 year-period, the HR variation observed during morning supine tests was reduced, with average daily values being 6 bpm higher in fatigue vs. non-fatigued states [32]. In addition, when investigating weekly values for HRV and HR, both measures were able to indicate non-functional overreaching with minimal differences, supporting the idea that weekly HR values are useful [18]. A valuable addition in this present study is the inclusion of HR during two different periods of rest. The HR values showed the highest level of agreement among morning and nocturnal values (Table 5), suggesting that the nocturnal HR values may be a valuable way to monitor resting HR. Previous findings found significant increases in nocturnal HR during a short period of intensified training and no changes in morning HR, suggesting that an improved accuracy may exist for nocturnal HR values [4]. In the current study, similar findings were observed with a significant decrease between the week 1 and week 3 nocturnal HR values, whereas the morning HR values displayed a decrease but at a slightly reduced level and without significance (Table 3). Similar to HRV, these differences may be due to external influences that are decreased during sleep and therefore, nocturnal HR values may provide a more observable relationship between HR and training status [31].

**Fig 3:**
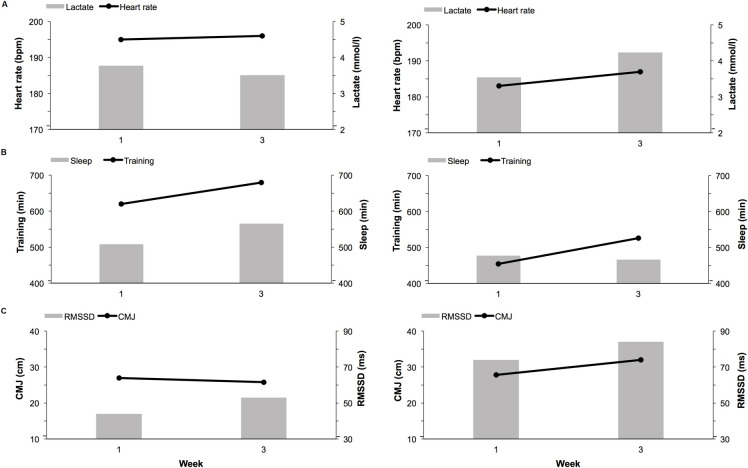
Comparisons of performance tests, nocturnal measures, and training results of two subjects during the study period. (A) Submaximal running test blood lactate and heart rate values during week 1 and week 3 for two different subjects. (B) Average values for night sleep and training during week 1 and week 3 for two different subjects. (C) Counter movement jump height and nocturnal RMSSD values during week 1 and week 3 for two different subjects.

We hypothesized that changes in HR and RMSSD would be associated to changes in SRT and CMJ tests. However, during the present study, training remained similar and no significant differences were present in SRT or CMJ, suggesting that the above changes are not due to changes in training load or volume (Table 3). Previous literature has suggested unloaded jumps are a common and useful way to monitor fatigue [4]. Research measuring weekly CMJ in distance runners found that jump performances the week before the season best competition were significantly higher than jump performance before the season worst completion [33]. In the present study, minimal changes between jump and performances tests, both as a group (Table 3) and on an individual level (Fig 3), during the test weeks illustrates that similar training was conducted during each test week and no significant differences were expected. However, a previous study with young athletes following a normal training protocol found submaximal HR values were associated with changes in performance variables over the entire season [5].

Additionally, during normal in-season training, good reliability was shown in both CMJ and SRT with a slight indication that SRT may be a more sensitive monitoring tool to predict performance changes [34]. Therefore, these findings imply that although no changes were found in our study, CMJ and SRT are acceptable tests to monitor young endurance athletes.

Our main concern in connection with our current investigation may be the lack of standardization of the measurement procedure, due to the collection of HR indices in individual home environments. Furthermore, although previous research has shown that resting HRV values do not differ between males and females [35, 36], the small sample size and grouping of gender are an additional limitation that should be noted. In addition, values calculated by the device may not provide the most reliable measures of HR indices since the accuracy of this device for continuous monitoring is yet to be determined. Since the extent of training stress also appeared to remain relatively constant, variation in the HRV was expected to be minimal. However, since our objective was to mimic typical every-day use, the subjects were instructed to maintain their routine patterns of sleeping and training.

## Conclusions

The present findings indicate that the accuracy and reliability of weekly average values for HR and RMSSD obtained during sleep employing a BCG-based device is acceptable. Thus, day-to-day monitoring of nocturnal HR and RMSSD appears to be a convenient and valid approach for long-term monitoring of young endurance athletes.

### Practical application

The application of BCG-based values used in the present study, provides facile monitoring of HR and RMSSD with a fully automatic and contact-free analysis without added time constraints [16]. Based on the data from this study, the nocturnal HR and nocturnal RMSSD values obtained appear to be a reliable way for coaches and athletes to monitor weekly average values. Therefore, long-term measurements are more attainable compared to morning measurements collected using electrodes or HR straps that require additional effort and thus, reduce daily athlete compliance [17]. It is important to note, that a practical monitoring system, especially for young athletes, should occur under relatively free-living conditions [31] with a balance between validity and ease-of-use in mind. Furthermore, the implementation of results is likely more effective when utilizing continuous measures that help identify individual variations rather than a less frequently collected value of slightly more power [2].
